# Getting Old through the Blood: Circulating Molecules in Aging and Senescence of Cardiovascular Regenerative Cells

**DOI:** 10.3389/fcvm.2017.00062

**Published:** 2017-10-06

**Authors:** Francesco Angelini, Francesca Pagano, Antonella Bordin, Vittorio Picchio, Elena De Falco, Isotta Chimenti

**Affiliations:** ^1^Department of Medical Surgical Sciences and Biotechnologies, “La Sapienza” University of Rome, Latina, Italy

**Keywords:** cell senescence, cardiac cell therapy, cardiovascular regeneration, insulin-like growth factor 1, endothelial progenitor cells, cardiac progenitor cells

## Abstract

Global aging is a hallmark of our century. The natural multifactorial process resulting in aging involves structural and functional changes, affecting molecules, cells, and tissues. As the western population is getting older, we are witnessing an increase in the burden of cardiovascular events, some of which are known to be directly linked to cellular senescence and dysfunction. In this review, we will focus on the description of a few circulating molecules, which have been correlated to life span, aging, and cardiovascular homeostasis. We will review the current literature concerning the circulating levels and related signaling pathways of selected proteins (insulin-like growth factor 1, growth and differentiation factor-11, and PAI-1) and microRNAs of interest (miR-34a, miR-146a, miR-21), whose bloodstream levels have been associated to aging in different organisms. In particular, we will also discuss their potential role in the biology and senescence of cardiovascular regenerative cell types, such as endothelial progenitor cells, mesenchymal stromal cells, and cardiac progenitor cells.

## Introduction

Aging is a natural multifactorial process of structural and functional changes, affecting molecules, cells, and tissues, therefore, representing a main risk factor for several clinical phenotypes, including cardiovascular diseases and chronic conditions (1). It is undeniable that the incidence of cardiovascular diseases, mainly heart failure, increases in the elderly population (2). Global aging is a hallmark of our century: the eldery population comprise roughly 15% of the population, and this scenario will increase of an additional 25% on average by 2050 (www.globalaginginstitute.org). This unprecedented population profile will inevitably imply, among others, an increasing burden of cardiovascular events, some of which are directly linked to cellular senescence and dysfunction. Thus, increasing knowledge on the various mechanisms causing the progressive decline of cellular and tissue function may aid in developing therapies to delay or treat age-related conditions and diseases, such as diabetes, cardiovascular and neurodegenerative diseases (3). Consequently, the discovery of pathways responsible for increasing life span and health span, as both potential biomarkers and targets, is currently of primary interest.

Since impairment in endogenous tissue function and repair is particularly exacerbated in the elderly persons and is involved in physiological aging and in chronic diseases (4), novel approaches of regenerative personalized medicine are being currently explored in order to ameliorate future therapeutic options for the aged society. Accordingly, several regenerative cell populations have been considered and tested in preclinical and clinical settings in the last years for cardiovascular applications, such as endothelial progenitor cells (EPCs) (5), mesenchymal stromal/stem cells (MSCs) (6), and resident cardiac progenitor cells (CPCs) (7, 8). Overall encouraging outcomes have been obtained in first clinical trials for several pathologies, such as revascularization strategies or cardiac cell therapy (5, 9), albeit with multiple issues still to be overcome (10).

Endothelial progenitor cells are considered a main circulating stem cell population finely controlling vascular homeostasis and repair in physiological conditions (11–14), therefore representing an interesting crossroad between circulating markers, regenerative cells, and aging mechanisms. According to their intrinsic property, EPCs represent *per se* a valuable biomarker for monitoring pathological states, in particular those associated with vascular damage. Importantly, the demonstration that EPCs can be systemically recruited from the bone marrow-associated niche, and that after engraftment are able to replace old vasculature with new mature endothelial cells, has completely overturned the theory about aging (11, 12, 15) and can be considered a significant reference for the relationship between progenitor cells and aging. To date, EPCs represent one of the most studied example tools to rejuvenate the vascular system or to potentially delay the damages induced by aging. Specifically, aging implies, among others, profound derangements in the endothelium and consequently in EPCs in terms of either number or function, directly altering their ability to generate new vessels (16). Similarly, growth factors and hormones modulate endothelial function. According to this vision, endothelial responsiveness in both healthy subjects and patients with cardiac conditions has been improved by antiaging strategies based on administration of growth hormone (GH), which, among its many functions, has been reported to increase the number of circulating EPCs (17, 18). More importantly, in aged organisms where inflammation is exacerbated due to a dysregulated production of soluble mediators, a reduction in the biological properties of EPCs is consequently found. Mechanisms underlying these alterations are still to be fully elucidated, but are considered critical to unravel the modality by which a boosted turnover of the endothelial system can be achieved. However, they are hypothesized to be different when aging is the natural consequence of a physiological process compared to induced or premature aging, as after a pharmacological treatment.

The other cell populations of interest for cardiovascular regenerative protocols, particularly cell therapy for heart failure, are CPCs and MSCs. Resident CPCs have been isolated form heart tissue in adult mammals, including humans, with several protocols, albeit only two approaches (i.e., spheroid culture selection as “cardiospheres” and CD117-sorting) have reached enough preclinical evidence to support clinical translation (7, 8). CPCs have been shown to support cardiac regeneration by direct differentiation toward cardiovascular lineages and paracrine effects (19, 20), and their biology and potency have been investigated in multiple settings (21, 22). MSCs have been introduced into clinical trials for cardiac cell therapy as well (23). They are mostly isolated from bone marrow and adipose tissue (24, 25), and contribute to heart regeneration mainly by paracrine mechanisms mediating cardioprotection and modulation of inflammation (26). Many clinical trials with MSCs have been completed and are currently ongoing for regenerative purposes, making them another important candidate for advanced cardiovascular therapies (23).

Notably, multiple studies suggest that, in the settings of cell transplantation for cardiovascular regenerative purposes, it is important not only to enhance intrinsic “young” properties of therapeutic cells, such as EPCs, but also to grant an ideal host microenvironment where engraftment can occur (27). Therefore, approaches able to rejuvenate regenerative cells and/or preserve tissue homeostasis and physiology (i.e., delaying overall aging) should be synergistically combined (16, 28).

One of the main mechanisms affecting senescence and aging at multiple levels is oxidative stress, which originates from several biochemical pathways triggered, among others, by environmental factors (29), and overall imbalancing the final amount of reactive oxygen species (30). Their strict association with aging and cardiovascular cell senescence has been already extensively overviewed elsewhere (31). In this review, we will discuss few circulating molecules [proteins and microRNAs (miRNAs)], selected among those whose levels and related signaling pathways have been correlated to life span and healthy aging. In particular, we will discuss pathways with specific biological and rejuvenating roles in cellular senescence, cardiovascular functions, and with a potential or known role in the phenotype control of regenerative cell populations.

## Insulin-Like Growth Factor 1 (IGF-1)

**Figure 1:**
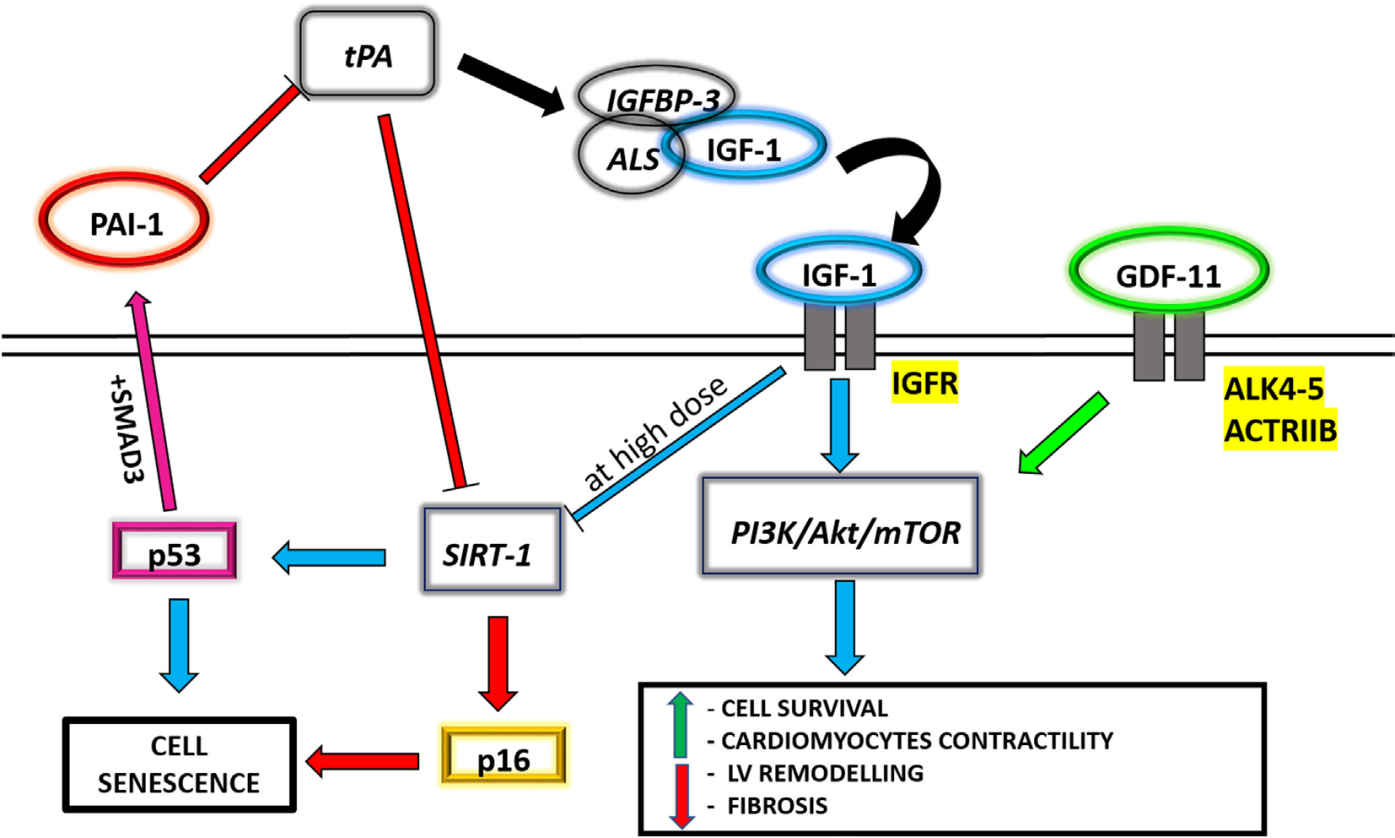
A schematic panel of the circulating molecules analyzed in the text, aiming at underlining the connections between their different related pathways.

Growth hormone (GH) and IGF-1, a circulating polypeptide hormone rather similar in molecular structure to insulin (Ins), are two key molecules involved in one of the most evolutionary preserved age-regulator-pathway. In 1957 Salmon and Daughaday postulated the so called “somatomedin hypothesis” suggesting that the GH, produced by the pituitary gland, was able to stimulate IGF-1 release by the liver, promoting its somatogenic actions in the target tissues (32). Subsequently, the discovery of local production of IGF-1 in many tissues, such as the heart, led to update the original theory, conferring also to this peptide important autocrine and paracrine functions (33). The IGF system, consisting of Ins, IGF-1, and IGF-2, is able to regulate many cell functions by binding specific transmembrane receptors (InsR, IGF-1R, IGF-2R) or binding proteins (IGFBPs) (34). Among these latter, IGFBP-3 in particular, in association with another protein, the acid-labile subunits, binds circulating IGF-1, prolongs its half-life from less than 5 minutes to 16 hours (35), and regulates its activity (36, 37) (Figure 1). In fact, this 150-kDa ternary complex serves as a potential reservoir of IGF-1 by sequestering the growth factor in the vascular compartment, and once reached the tissues, IGF-1 is released from the complex by proteolysis of IGFBP-3 (38). It is well known that the secretion of GH and IGF-1 declines progressively to very low levels with aging, a phenomenon called “somatopause” (39), and this evidence has suggested it as an hypothetical “longevity pathway” able to modulate aging processes (40).

Regarding the cardiovascular system, IGF-1 directly cooperates with the phosphatidylinositol 3-kinase (PI3K)/Akt/mechanistic target of rapamycin (mTOR) signaling pathway, thus modulating the adaptive response of cardiac muscle to altered conditions of hemodynamic overload, also known as physiological hypertrophy (41). The PI3K/Akt/mTOR pathway is related to Ins sensitivity and calorie restriction (CR), which is the reduction of total caloric intake with an adequate balanced nutrition. CR is among the most well-known conditions able to increase life span and health span in different animal models (42). Since the PI3K/Akt/mTOR pathway is considered central in aging and Ins control (43), this strengthens the concept that endocrine factors, able to directly interact and coordinate PI3K/Akt/mTOR signaling, could regulate longevity. It has been demonstrated that IGF-1 induces an increase in cardiomyocyte size, with preserved systolic function and absence of fibrosis, and upregulates the expression of cardiac specific genes, such as troponin I and myosin light chain-2v (44, 45) (Figure 1). The same effect, in terms of attenuation of left ventricular remodeling and enhancement of Akt signaling, is also indirectly promoted by an IGF-1-dependent myocardial SERCA2 content improvement (46). Regardless of these evidences, the mechanisms that link IGF-1, aging, and cardiovascular diseases are still debated. Several population studies have correlated an increased risk for all-cause mortality (including cardiovascular) to the presence of low circulating IGF-1 levels (47, 48). Analysis carried out on a specific exceptionally long-living human population, though, have associated high IGF-1 levels found in centenarians with a compensatory effect due to genetic variants with reduced activity in IGF-1R signaling (49, 50), suggesting that high IGF-1 levels in elderly persons may be, at least in part, a compensatory mechanism. Nonetheless, Tran et al. (51) have suggested a molecular connection between IGF-1 and the sirtuin-1 (SIRT-1)/p53 pathway, responsible for inducing cell senescence (Figure 1). SIRT-1 is a well-known NAD-dependent deacetylase involved in aging. In fact, sirtuins are crucial during cell response to stress and for cell metabolism regulations, and their increasing levels, due to a proper lifestyle, are able to influence health span and, possibly, life span (52). Likewise, the role of p53 in promoting cellular senescence in response to cellular stress is also well demonstrated (53) by means of its inhibition through SIRT-1 deacetylation (54). It has been observed *in vitro* that IGF-1 treatment on human and murine primary fibroblasts is able to promote cell proliferation and survival in the short period, while a prolonged administration of IGF-1 inhibits SIRT-1 deacetylase activity, increasing p53 acetylation and activation and leading to premature cell senescence (51).

Insulin-like growth factor 1 is known to have multiple effects on cardiovascular regenerative cells, such as resident CPCs. As previously mentioned, IGF-1 counteracts senescence and apoptosis in the heart (55), with the ability also to support the resident CPC pool (56) and enhance their homing in animal models of therapeutic protocols (57). Other than responding to IGF-1, CPCs release IGF-1 in an autocrine/paracrine way (19), thus potentially contributing to endogenous anti-senescent signals. When CPCs are cultured in 3D spheroid systems, mimicking the *in vivo* stem cell niche (58, 59), IGF-1 release is increased (19), and a highly controlled production of proteases (e.g., PAPP-A) against IGFBPs occurs, able to locally increase IGF-1 bioavailability (60). Moreover, GH-induced IGF-1 increase has been shown *in vivo* to significantly attenuate cellular senescence of EPCs (61), which are considered *per se* as a circulating index of aging and impaired cardiovascular function (62). Overall, these results highlight an interesting relationship between IGF-1, as a circulating antagonist of aging, and its positive effects on the biology of cardiovascular regenerative cell types. We can speculate that this effect may be one of the beneficial mechanisms sustaining long lasting tissue homeostasis associated to high circulating IGF-1 levels in long-living healthy mammals.

## Growth and Differentiation Factor-11 (GDF-11)

Another important and debated aging-related circulating factor, belonging to the transforming growth factor-β (TGF- β) superfamily, is the GDF-11 that is highly conserved in several species, including human, mice, rat, and invertebrates (63). The TGF-β superfamily has been divided into three main subclasses: TGF-βs, bone morphogenetics proteins, and activin/myostatins (MSTNs) (64). GDF-11 belongs to the activin/MSTNs subclass and regulates gene expression through phosphorylation of the signal transductors SMAD2 and SMAD3 (64). This complex migrates and accumulates in the nucleus to regulate gene expression through direct and indirect DNA binding (65, 66). Furthermore, several non-SMAD pathways have been discovered, such as PI3K/Akt/mTOR signaling, by which GDF-11 regulates a variety of cellular functions (67) (Figure 1). Even if it is well known that GDF-11 is involved in mesoderm formation and neurogenesis during embryonic development (68, 69), its role in postnatal tissues is less clear and still under investigation. It has been described as a mediator of aging processes in multiple tissues, particularly heart, brain, and muscle (70). GDF-11 role in aging in the cardiovascular system was firstly investigated in 2013 by Loffredo et al. At first, they underlined an evident age-dependent decrease in circulating GDF-11 levels, and they cataloged this protein as a “pro-youthful” factor based on a parabiosis experiment (71). In fact, after exposing old mice to the circulation of young ones, they observed, through heart weight measurements, morphometric, and molecular analyses, a regression of cardiac hypertrophy levels comparable to those found in a young animal. However, in another study of daily injections of recombinant GDF-11 in old mice with the rationale to restore the level of this protein to youthful levels, the authors did not observe any improvement in the overall heart size, in cardiac myocytes volume, or any changes in cardiac performance (72). In the last years, many studies have investigated GDF-11 role on cardiac aging and remodeling, demonstrating the pros and cons of the “rejuvenating theory” in humans and other animal models (73–75), proving that, despite the controversy, this protein represents nonetheless a “hot spot” for understanding aging mechanisms and how to ameliorate life and health span.

Concerning the specific *in vivo* role of GDF-11 on cardiovascular regenerative cells biology, it has been shown that targeted myocardial delivery of GDF-11 gene to the aged heart after ischaemia/reperfusion restored GDF-11 expression and contained tissue damage. GDF-11 levels were also associated to reduced cell senescence markers, increased proliferation of Sca-1+ CPCs, and increased homing of EPCs with angiogenesis in old ischemic hearts (76). Moreover, GDF-11 has been reported to be a key regulator of stem/progenitor cells phenotype and stemness control in multiple tissues, such as muscle and brain, where it is able to control proliferation, activation, and function of muscle satellite cells and neural stem cells, respectively (70). It is worth mentioning that skeletal myoblasts and satellite cells have been tested in the past as therapeutic cell products for cardiac cell therapy, although their clinical translation has been abruptly stopped due to dangerous arrhythmogenic side effects (22). A study by Finkenzeller et al., instead, has demonstrated *in vitro* that GDF-11 improves vasculogenesis by activating the Smad2/Smad3 pathway using EPCs isolated from peripheral blood (pbEPCs) (77). Accordingly, future studies will be needed to provide further insights on the possible role of GDF-11 on the phenotype and senescence of cardiovascular regenerative cell types, including MSCs.

## PAI-1

The circulating factors discussed until now have in common a negative correlation between their plasma concentration and increased age, and this defines them as possible, albeit partly debated, “rejuvenating” agents and/or aging biomarkers. However, it has been reported the existence of other important circulating factors which increase during aging, and could conversely represent an anti-aging target. Fibrinolysis, the proteolytic degradation of fibrin clots, is mediated by plasmin, which is formed through the activation of its precursor plasminogen by urokinase-type plasminogen activator and tissue-type plasminogen activator (t-PA) (78). The dynamic equilibrium of the fibrinolytic process is regulated also by the presence of specific inhibitors, such as alpha-2-antiplasmin and plasminogen activator inhibitors (PAIs). These latters represent a family of serine-protease inhibitors, also known as serpins, composed by three types of PAI: PAI-1, PAI-2, and PAI-3 (79). In particular, plasma circulating factor PAI-1, despite its short half-life of around 2 hours (80), contributes substantially not only to fibrinolysis but also to a variety of biological processes, such as pericellular proteolysis, cell adhesion and migration, cell–matrix interactions, and signaling pathways (81–83).

High PAI-1 levels are involved in a number of age-related subclinical and clinical conditions, including Ins resistance and cardiovascular disease (84), as demonstrated analyzing several animal models of aging and/or population studies (85, 86). Recently, it has become evident that PAI-1 is synthesized and secreted by senescent cells and plays a critical role in the regulation of aging. In fact, it has been demonstrated that PAI-1 is able to inhibit the proteolysis of IGFBP-3 by blocking t-PA, reducing accordingly IGF-1 release and inducing an accumulation of the inhibitor of cyclin-dependent kinases p16, leading to G1 cell cycle arrest and cellular senescence (87, 88) (Figure 1). Furthermore, Kortlever et al. have demonstrated that p53, in association with SMAD3, is able to upregulate PAI-1 expression in aging mice and human fibroblasts, causing a downregulation of PI3K signaling and cyclin nuclear exclusion (89, 90) (Figure 1). These findings were reinforced by Gosh et al. through a small molecule, called TM5441, which is a potent inhibitor of PAI-1. Indeed, they demonstrated that the pharmacological inhibition of PAI-1 has protective effect on aging-induced cellular senescence in cardiac myocytes, fibroblasts, and endothelial cells, suppressing specific regulators such as p16 and p53 (91).

Concerning cardiovascular regenerative cells, it has been recently demonstrated that kallistatin, a protein known to counteract vascular senescence, exerts its anti-senescent effects also through PAI-1 downregulation in EPCs, opposing TNF-α-induced cellular signaling (92). Interestingly, increased PAI-1 levels have been also shown to negatively affect the survival and anoikis resistance of therapeutic MSCs in ischemic-like conditions (93), which is a main pathophysiological event in many cardiovascular diseases. Overall, these few studies suggest that targeting PAI-1 levels may be a strategic approach, among others, also to boost regenerative approaches.

## Circulating miRNAs

Among the circulating factors associated with aging, there are several miRNAs (94); these are small regulatory RNAs that can act as intracellular regulators or be secreted, and that mediate intercellular communication also through cellular junctions (95). These molecules can be released in the blood flow and are exceptionally stable in body fluids, including serum and plasma (96). Circulating miRNAs can be embedded into protein complexes (Ago2, Nucleophosmin-1), linked to lipoproteins such as high density lipoproteins, or within exosomes, microvescicles, and apoptotic bodies (97). These complexes protect the RNA from RNAse degradation and make circulating miRNAs eligible candidates as stable biomarkers and mediators for many pathological conditions, including cardiovascular diseases (98). A very recent review from Olivieri et al. discusses the most recent papers on the profiling of circulating miRNAs during aging in animal models, as well as human cohorts, providing a comprehensive review of the topic (99). Here, we will focus on few circulating miRNAs, which have been correlated to longevity (100, 101), and which have also a specific role in the biology of cardiac, vascular, and circulating progenitor cells.

Physiological aging can influence the amount of specific circulating miRNAs, as shown by studies in animal models and through comparison of elderly versus younger subjects (102), and some of them have a role in cardiovascular system biology and physiopathology (98). In line with several reports describing the effect of the microenvironment generated in the aging organism, a recent retrospective study on the circulating miRNome of twins has shown differences in circulating miRNAs levels as the twins age. Comparison of deceased twins with their alive co-twin brothers has showed that the profile and concentration of specific circulating miRNAs was different, and the different life span was reflected in a very dissimilar expression of the majority of miRNAs. This suggests that environmental factors are crucial for the presence of life expectancy-related circulating miRNAs (103).

**Figure 2:**
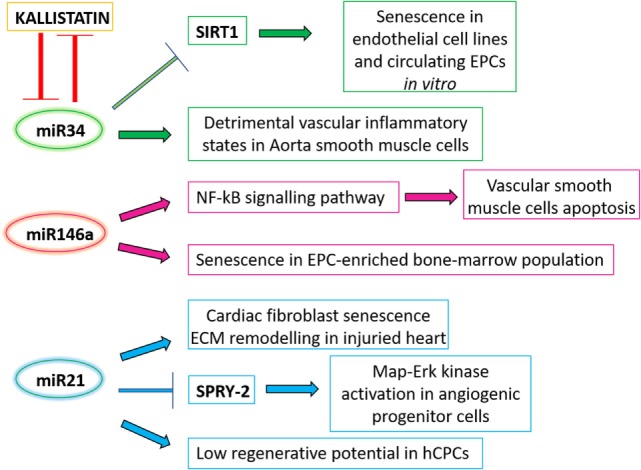
A schematic panel of the circulating microRNAs analyzed in the text and their related effects. ECM, extracellular matrix.

The presence in the blood stream of miR-34 family members (miR-34 b/c) correlates with age in animal models (104), as well as in elderly subjects compared to younger (101, 105) (Figure 2). SIRT-1 is a well-studied miR-34-family target gene, and the miR-34-mediated downregulation of SIRT-1 has been shown to induce senescence in both endothelial cell lines and circulating EPCs *in vitro* (106, 107). In addition, miR-34 expression is increased in aorta smooth muscle cells during mice aging, and its high expression leads to the release of pro-inflammatory secretory factors from these cells (108), therefore contributing to detrimental vascular inflammatory states observed in aged organisms. Interestingly, miR-34a downregulation is mediated by the previously mentioned anti-senescent molecule, kallistatin, while consistently miR-34a overexpression is able to abolish kallistatin’s anti-senescent activity in EPCs (92). Finally, IGF-1 can specifically block the expression of the precursor and mature miR-34a, while consistently miR-34a overexpression is able to abolish the antiapoptotic effect of IGF-1 (109). Overall this data support a role for this miRNA ranging from systemic circulating levels to local vascular and homeostatic effects.

A correlation between circulating miRNA levels and inflammatory status has been also addressed in a study of genotype-by-age interaction in mice, which has revealed a peculiar miRNA pattern in the blood of animals with different genotypes as they age. Specifically, miR-146a is increased in the aging control mice, but remains unchanged in the long-lived hypopituitary Ames dwarf mice (104), which in addition show reduced meta-inflammation (110). Concerning its effects on cardiovascular and regenerative cells, a direct effect of miR-146a on vascular cells’ survival has been described in animal models, where miR-146a overexpression induced vascular smooth muscle cells apoptosis through activation of the NF-KB signaling pathway (111) (Figure 2). Interestingly, features of increased functional cell yield and cardiogenic potential in CPCs have been associated to reduced levels of miR-146a (112). Moreover, miR-146a overexpression has been shown to increase cell senescence in an EPC-enriched bone marrow cell population, impairing their angiogenic properties (113).

The levels of circulating miR-21 have been associated with longevity (105, 114): in fact, its downregulation is associated with healthy aging. This miRNA has many functions within the cardiovascular system; it has been described to induce cardiac fibroblasts senescence and extracellular matrix remodeling in the injured heart (115–117) (Figure 2). A recent *in vitro* study on human circulating angiogenic progenitor cells (APCs) showed an effect of miR-21 increased expression in these adult cells, which are involved in vascular regeneration and homeostasis, as do EPCs. Overexpression of miR-21 is able to mediate APCs dysfunction through downregulation of superoxide dismutase 2, and activation of Map–Erk kinase pathway through SPRY2 inhibition. This resulted in impairment of nitric oxide availability and APC dysfunction (118), which ultimately might lead to lower reparative and regenerative capabilities. Consistently, increased miR-21 expression levels have been also associated to features of reduced cardiovascular regenerative potential in human CPCs (112). Overall these studies suggest that, together with circulating proteins, epigenetic mediators may play a key role in physiologic aging mechanisms in the cardiovascular system, affecting local regenerative potential.

## Conclusion

In this review, we aimed at suggesting and discussing circulating molecules and related pathways in aging, whose understanding may potentially lead to the identification of diagnostic, prognostic, or even therapeutic targets, albeit the available data do not allow definitive clinical stands yet. As discussed, multiple circulating factors, ranging from proteins to RNAs to actual cells (i.e., EPCs), can be considered as aging mediators that offer interesting clues on senescence mechanisms and with potential diagnostic and therapeutic implications (Figures 1 and 2). Many of these factors have been associated with reduced regenerative potential and increased senescence of cell populations which are under preclinical and/or clinical investigation as cell product candidates for cardiovascular cell therapy protocols. Increasing knowledge on the relationship between circulating mediators and the pathways they may modulate (at both systemic and local level) may provide novel insights for the optimization and improvement of cardiovascular personalized regenerative medicine in the aging population.

## Author Contributions

IC and EF: conception and design of the work; IC, EF, FA, FP, AB, and VP: drafting of the work, data acquisition, and analysis of the bibliography; IC, EF, and FA: final approval of the version to be published.

## Conflict of Interest Statement

The authors declare that the research was conducted in the absence of any commercial or financial relationships that could be construed as a potential conflict of interest.
